# UHPLC-HRMS Analysis of *Fagus sylvatica* (Fagaceae) Leaves: A Renewable Source of Antioxidant Polyphenols

**DOI:** 10.3390/antiox10071140

**Published:** 2021-07-19

**Authors:** Marialuisa Formato, Simona Piccolella, Christian Zidorn, Severina Pacifico

**Affiliations:** 1 Department of Environmental, Biological and Pharmaceutical Sciences and Technologies (DiSTABiF), University of Campania ‘Luigi Vanvitelli’, Via Vivaldi 43, 81100 Caserta, Italy; marialuisa.formato@unicampania.it (M.F.); simona.piccolella@unicampania.it (S.P.); 2 Department of Pharmaceutical Biology, Pharmaceutical Institute, Kiel University, Gutenbergstraße 76, 24118 Kiel, Germany; czidorn@pharmazie.uni-kiel.de

**Keywords:** *Fagus sylvatica* L., beech leaf, ultra-high-pressure liquid chromatography electroSpray ionization quadrupole time-of-flight mass spectrometry (UHPLC-ESI-Q*q*TOF HRMS) analysis, antioxidant assays, polyphenol recovery

## Abstract

European beech (*Fagus sylvatica* L.) is a deciduous tree, widely distributed in Europe and largely appreciated for its wood and nutritive nuts. Beech leaf also enjoys food use as salad, but an understanding of its nutraceutical value is still far from being achieved. Indeed, and also taking into account beech leaf as a consistent biomass residue available beechwood production and use, it needs to be explored as a valuable renewable specialized source of bioactive molecules. In this context, an untargeted ultra-high-performance liquid chromatography hyphenated with high resolution mass spectrometry (UHPLC-HRMS) approach was favorably applied to a beech leaf alcoholic extract, which also was evaluated for its antiradical capability (by means of assays based on 2,2-diphenyl-1-picrylhydrazyl (DPPH) and [2,2’-azinobis-(3-ethylbenzothiazolin-6-sulfonic acid)] (ABTS) radical cation) and its ferric ion reducing power. Redox mitochondrial activity towards Caco-2 cells paved the way to explore the extract’s capability to inhibit intracellular Reactive Oxygen Species (ROS) using 2’,7’dichlorofluorescin diacetate (DCFH-DA) assay. Hydroxycinnamoyl derivatives, mainly belonging to the chlorogenic acid class, and flavonoids were the main constituents. Uncommon flavanone *C*-glycosides were also found, together with a plentiful flavonol diversity. Cell-free and cell-based assays highlight its dose-dependent antioxidant efficacy, providing a foundation for further investigation of beech leaf constituents and its valorization and use as a reservoir of bioactive natural products with potential nutraceutical applications.

## 1. Introduction

Nowadays, the replacement of synthetic and artificial chemicals with natural products with less impact on human or animal health and environment, makes their recovery a major challenge. Indeed, great efforts have been devoted to the discovery and exploitation of renewable sources of valuable bioactive compounds, such as the so-called bioactive specialized natural products. These latter include a number of compound classes and sub-classes that, in the last years, have attracted a lot of attention for a possible application in various sectors (i.e., nutri-cosmeceutical, medical and pharmacological), due to their recognized benefits for human and animal health [1]. Thus, the research and valorization of new plant matrices, also those not directly used for these purposes, need to be explored as a virtuous source of these molecules. Innovative examples are agro-food wastes [2,3,4], or biorefining of forest biomass [5], from which compounds with traditional use in the prevention and/or treatment of different diseases could be favorably isolated [6]. By-products from forestry and wood processing industry are promising feedstocks for the extraction of polyphenols, including tannins and other antioxidants [7]. Thus, the self-renewal of plant matrices such as the deciduous leaves of forest or ornamental trees, could be an alternative reservoir of compounds, exploitable for different useful applications.

Among forest trees, the Fagaceae family includes more than 900 species belonging to 8–10 genera, among which only *Castanea*, *Fagus* and *Quercus* are distributed in Asia, Europe and North America. In particular, *Fagus sylvatica* L., known as common or European beech, is an economically important tree species. It is widely distributed, from Northern Spain to Southern Italy and the North of Greece in the South Europe up to Southern Scandinavia in the North, and from the Atlantic Ocean in the West to the Black Sea in the East. 

*F. sylvatica* leaves, as well as those of other forest trees, played an important role in winter-feeding of livestock in Europe even after haymaking replaced leaf-fodder harvesting over time. Indeed, different organs of *Fagus sylvatica* could be used as source of several natural products [8]. Thus, while beech bark and wood could be a source of different polyphenols [9], the fruits, named beechnuts, are used to obtain an oil rich in oleic and linoleic acids, as well as in γ- and δ-tocopherols [10]. Unlike wood, beechnuts or bark, whose chemical composition was rather well studied, there is very little literature data on the chemical composition of the leaves [11], which comprises phenols and polyphenols such as flavonoids, hydroxycinnamic acids and procyanidins [8,12], and triterpenes, such as glycosylated oleanane-type saponins [13]. Leaf ethanolic extracts showed antimicrobial activity [14], gastroprotective effects [15], with efficacy against *Helicobacter pylori*, and anti-proliferative properties [16]. Beyond their chemical composition and biological activity, leaves are considered an index to evaluate environmental pollution and defensive mechanism to stress [17]. In the light of the above, the main objective of this study was to gain deep insights into the polyphenol composition of *F. sylvatica* leaves, in order to show the potential of their exploitation to obtain bioactive molecules. For this purpose, an untargeted UHPLC-HRMS (Ultra-High-Performance Liquid Chromatography-High-Resolution Mass Spectrometry) approach was applied to a methanolic leaf extract. The determination of total phenols and flavonoids, as well as the antiradical and reducing activity of the extract was also assessed, while its ability to slow down intracellular reactive oxygen species (ROS) formation was preliminarily investigated in Caco-2 cells.

## 2. Materials and Methods

### 2.1. Plant Collection and Extraction 

*Fagus sylvatica* leaves were collected in June 2017 in the Tannenberger Gehölz (Kiel, Schleswig-Holstein, Germany, N 54°21′52.6′′, E 10°06′35.9′′, 25 m a. m. s. l., Google Earth). In particular, leaves harvested between ca. 0.5 and 2 m above ground were collected from six individual trees of various ages. The month average precipitation was 5 mm and average temperature was 21–10 °C (max–min). Voucher specimens were deposited in the herbarium of Kiel University (KIEL, Schleswig-Holstein Land, Germany) and the private herbarium of Christian Zidorn (voucher code: FS_20160705A-1). Air-dried leaves (240 g) were shredded, and then extracted by maceration in methanol overnight, followed by an ultrasound accelerated maceration step (UAM; Bransonic^TM^ M3800-E, Branson Ultrasonics^TM^, Danbury, CT, USA) (three sonication cycles, 35 min each). The alcoholic extract was stored at −20 °C until use.

### 2.2. UHPLC-HRMS and MS/MS Parameters 

The alcoholic extract was investigated using a NEXERA UHPLC system (Shimadzu, Tokyo, Japan) equipped with a Luna^®^ Omega C-18 column (50 × 2.1 mm i.d., 1.6 µm particle size). Two µL of the sample were injected. The mobile phase was constituted by water (solvent A) and acetonitrile (solvent B), both acidified with formic acid (0.1% *v*/*v*). 

A linear gradient was used, in which the percentage of solvent B increased as follows: 0–5 min, 5%→15% B; 5–10 min, 15% B; 10–12 min, 15%→17.5% B; 12–17 min, 17.5%→45%B; 17–18.50 min, 45% B; 18.51–20 min, column re-equilibration. The flow rate was set at 400 µL/min. 

High-Resolution Mass Spectrometry (HR-MS) data were obtained by an AB SCIEX Triple TOF^®^ 4600 mass spectrometer (AB Sciex, Concord, ON, Canada), equipped with a DuoSpray^TM^ ion source (AB Sciex, Concord, ON, Canada) operating in the negative ElectroSpray (ESI) mode. A full scan Time-Of-Flight (TOF) survey (accumulation time 100 ms, 100–1000 Da) and 8 information-dependent acquisition MS/MS scans (accumulation time 50 ms, 80–850 Da) were acquired, using the following parameters: curtain gas 35 psi, nebulizer and heated gases 60 psi, ion spray voltage 4500 V, ion source temperature 600 °C, declustering potential −70 V, collision energy −35 ± 5 V. The instrument was controlled by Analyst^®^ TF 1.7 software (AB Sciex, Concord, ON, Canada), whereas MS data were processed by PeakView^®^ software version 2.2 (AB Sciex, Concord, ON, Canada). The compounds were identified mainly through the study of their tandem mass spectrometry (TOF-MS/MS) fragmentation patterns, and the comparison with literature data whenever possible.

### 2.3. Radical Scavenging Capacity: DPPH and ABTS Tests

Leaf alcoholic extract was tested at 200, 100, 50, 25, 12.5, 6.25, and 3.125 μg/mL (final concentration levels) towards ABTS [2,2′-azinobis-(3-ethylbenzothiazolin-6-sulfonic acid)] radical cation and 2,2-diphenyl-1-picrylhydrazyl (DPPH) radical. 

ABTS radical cation was generated as previously reported [18]. The ABTS^●+^ solution was diluted with Phosphate-buffered saline (PBS; pH 7.4) until an absorbance of 0.7 at 734 nm was read. The extract at different doses was directly dissolved in the ABTS^●+^ solution, and after 6 min the absorbance was measured by a Victor3 spectrophotometer (Perkin Elmer/Wallac, Waltham, MA, USA) in reference to a blank, in which the samples were replaced with solvent [18].

DPPH^●^ scavenging capability was estimated as previously reported [18], and the absorption at 517 nm was measured on the Victor3 spectrophotometer (Perkin Elmer/Wallac, Waltham, MA, USA) in reference to a blank, in which the samples were replaced with the solvent. 

Trolox (4, 8, 16, 32 µM) was used as positive standard, and Trolox Equivalent Antioxidant Capacity (TEAC) of beech-leaf extract was calculated, based on both ABTS and DPPH tests. For each antiradical test, three replicate measurements for three samples (*n* = 3) of the extract (in total, 3 × 3 measurements) were performed. All data were expressed as mean ± standard deviation (SD).

### 2.4. Fe(III) Reducing Power

Beech leaf alcoholic extract (at 200, 100, 50, 25, 12.5, 6.25, and 3.125 μg/mL final concentration levels) was investigated for its ability to reduce the Fe^3+^ using ferricyanide FRAP assay, as previously reported [19]. The absorbance was measured at 700 nm. The increase in absorbance with reference to the blank was considered to value the reducing power. Trolox (4, 8, 16, 32 µM) was used as positive standard, and TEAC value of beech-leaf extract was calculated. The test was carried out performing three replicate measurements for three samples (*n* = 3) of the extract (in total, 3 × 3 measurements). All data were expressed as mean ± standard deviation (SD). 

### 2.5. Determination of Total Phenols

Total phenol content was determined according to the Folin-Ciocalteau procedure [19]. Samples (0.25 mg and 0.125 mg) were mixed with 2.25 mL of Na_2_CO_3_ (7.5% *w*/*v*) and 0.25 mL of Folin-Ciocalteu reagent. The tubes were mixed and allowed to stand for 3 h at room temperature. The absorbance was read at 765 nm using a Synergy spectrophotometer (Biotek, Winooski, VT, USA). The test was carried out performing three replicate measurements for three samples (*n* = 3) of the extract (in total, 3 × 3 measurements). Data were expressed as milligrams of gallic acid equivalents (GAEs) per g of extract (mean ± standard deviation). To this purpose, a gallic acid calibration curve (R^2^ = 0.9716) was built up in the range 0.78–25 μg/mL (final concentration levels).

### 2.6. Determination of Total Flavonoids

The extract (0.5 mL) was dissolved in distilled water (5 mL), and NaNO_2_ solution (5%, *w*/*v*; 0.3 mL) was added. After 5 min, AlCl_3_ solution (10%, *w*/*v*; 0.6 mL) was poured into the flask, and after 6 min, NaOH solution (1.0 M; 2.0 mL) and distilled water (2.1 mL) were added. The absorbance was read at 510 nm against the blank (water), and flavonoid content is expressed as milligrams of quercetin equivalents per 100 g of fresh material [20]. To this purpose, a quercetin calibration curve (R^2^ = 0.9979) was built up in the range 0.78–100 μg/mL (final concentration levels). The test was carried out performing three replicate measurements for three samples (*n* = 3) of the extract (in total, 3 × 3 measurements). All data were expressed as mean ± standard deviation (SD). 

### 2.7. Cell Culture, Cytotoxicity and Intracellular ROS Assessment

Human epithelial cell line Caco-2 (ATCC^®^ HTB¬37™, American Type Culture, Manassas, VA, USA) was cultured in Dulbecco’s Modified Eagle’s Medium (DMEM) supplemented with 10% fetal bovine serum, 50.0 U/mL of penicillin and 100.0 μg/mL of streptomycin, at 37 °C in a humidified atmosphere containing 5% CO_2_. Cells were seeded in 96-multiwell plates at a density of 2.5 × 10^4^ cells/well. After 24 h, cells were treated for 24 h with different doses of the beech leaf alcoholic extract (25, 50, 100 and 200 µg/mL) or pure quercetin standard (5, 10, and 50 µM). When incubation was completed, inhibition of mitochondrial redox activity was determined by the MTT cell viability test, which was based on the 3-(4,5-dimethylthiazol-2-yl)-2,5-diphenyltetrazolium bromide (MTT) dye, as previously described [3,4]. In order to evaluate intracellular ROS inhibition [21], Caco-2 cells were seeded at a density of 2.5 × 10^4^ well on a black 96-well microplate in 100 μL growth medium/well. Cells were cultured for 24 h at 37 °C in 5% CO_2_ and observed under the inverted phase contrast microscope. After 24 h, the growth medium was removed, and cells were twice washed with PBS (100 μL). Then, the cells were co-exposed to the investigated extract (25, 50, 100 and 200.0 µg/mL; final concentration levels) or quercetin (10 μM) and 2′,7′-dichlorofluorescin diacetate (DCFH-DA; 60 μM) for 60 min. The treatment medium was then removed, the cells were washed with PBS, and 2,2′-azobis(2-methyl propionamidine)dihydrochloride (AAPH, 500 μM; 100 μL) was added. The 96-well microplate was placed into a PerkinElmer Victor3 Multilabel Plate Reader at 37 °C. The fluorescence intensity was measured at 485 nm excitation and 535 nm emission wavelength every 20 min for 100 min. Two independent experiments were carried out performing in each six replicate measurements for three samples (*n* = 3) of the extract (in total, 6 × 3 measurements). Data were expressed as mean ± standard deviation (SD).

## 3. Results and Discussion

### 3.1. Chemical Composition of F. sylvatica Leaf Methanolic Extract

Nowadays, the growing consciousness of environmental sustainability promotes the recovery of waste from production chains, also as basis and foundation of a circular economy. Renewable forest materials are rich in nutrients and bioactive molecules, whose recovery opens up to the development of functional products with high added value [22], to be used in various production sectors, from food, to nutraceuticals, cosmetics, up to the creation of packaging. In this context, getting insight into the chemical composition of beech leaf, its diversity in polyphenols could represent its intrinsic value. 

The analytical determination of the diversity in bioactive molecules, after suitable extraction of the matrix, represents the crucial step of the entire process. Actually, techniques in tandem mass spectrometry hyphenated with chromatographic separation methods allows the chemical composition to be finely unraveled through an untargeted approach that provides accuracy in phenolomic data and compounds identification [23]. 

**Figure 1:**
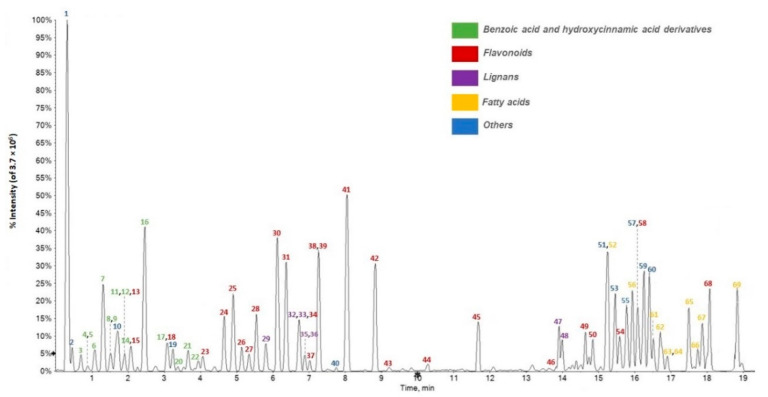
UHPLC-ESI-Q*q*TOF MS profile (Total Ion Chromatogram acquired in negative ion mode) of *F. sylvatica* leaf extract under study. Compounds were numbered, based on their increasing retention time (RT).

In the present study UHPLC-ESI-HRMS and High-Resolution tandem mass spectrometry (HR-MS/MS) techniques were first applied to unravel the chemical composition of this undervalued plant source [24,25,26]. An untargeted metabolic approach was used. Sixty-nine compounds were tentatively identified (Figure 1), mainly belonging to phenol and polyphenol classes. These latter have been described also as constituents of other forest trees and their wastes. Indeed, recently, bulk samples of bark waste from *Pinus contorta*, *Pinus sylvestris*, and *Quercus robur*, were investigated in depth for their polyphenol content [27], and various biomass residues (shavings, edged cuts, and pruning wastes) from walnut were analyzed as sources of antioxidant compounds by means of a green extraction process [28]. In this context, Pycnogenol^®^ (PYC) and Flavangenol^®^ are good examples of commercially available pine bark-based products [29]. The bark of douglas fir (*Pseudotsuga menziesii* Franco), one of the premier timber trees in the world, was found a rich source in taxifolin, which is broadly applied in pharmaceutical preparations [30].

In Table 1, ESI negative ion mode MS and MS/MS data, molecular formulas, unsaturation degree (RDB-Ring and Double Bond) values and mass accuracy are listed. 

#### 3.1.1. Benzoic and Hydroxycinnamic Acids Derivatives

Compounds **4**, **5** and **6** were tentatively identified as (di)hydroxybenzoic acid hexosides and dihydroxybenzoic acid, respectively. In fact, deprotonated glycosides underwent homolytic and heterolytic cleavages of the hexose moiety, providing fragment ions at *m*/*z* 153.0191 and *m*/*z* 152.0112. 

**Figure 2:**
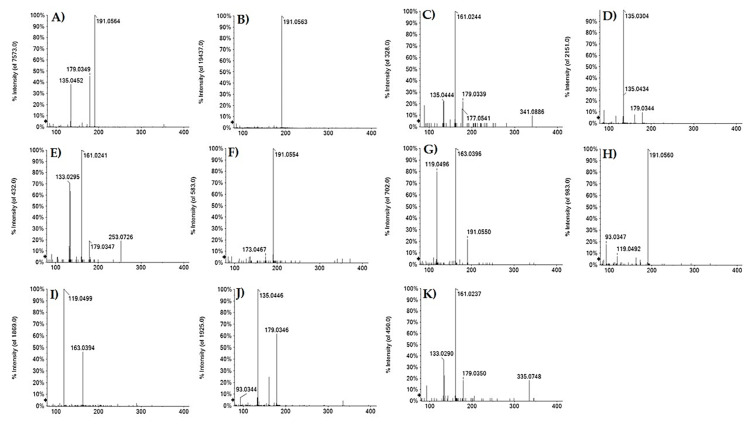
TOF-MS/MS spectra of deprotonated compounds (**A**) **7**; (**B**) **16**; (**C**) **8**; (**D**) **9**; (**E**) **17**; (**F**) **3**; (**G**) **11**; (**H**) **20**; (**I**) **14**; (**J**) **21**; (**K**) **22**.

Compounds **7** and **16**, previously identified in *F. sylvatica* leaves, were tentatively identified, in a ratio 1:2, as 3-*O*- and 5-*O*-caffeoyl quinic acid (Figure 2, panels A and B) based on the elution order and different fragmentation patterns [31]. The fragment ion at *m*/*z* 179.03 (deprotonated caffeic acid) was also identified in TOF-MS/MS spectra of compounds **8** (at *m*/*z* 341.0879), **9** (at *m*/*z* 297.0613) and **17** (at *m*/*z* 253.0718), which were tentatively identified as caffeoyl acid hexoside (C_15_H_18_O_9_), caffeoyl threonic acid (C_13_H_14_O_8_) and caffeoyl propanoic acid (C_12_H_14_O_6_), respectively. The fragmentation pattern of metabolite **9** showed also product ions at *m*/*z* 135.0304 and *m*/*z* 117.0192 (Figure 2D), corresponding to deprotonated threonic acid and its dehydrated derivative. Instead, for metabolite **17** the propionyl moiety (C_3_H_6_O_2_) was identified by neutral loss of 74.04 Da (Figure 2E). Other caffeoyl derivatives were the metabolites **3**, tentatively identified as hydroxycaffeoyl quinic acid, and **12**, that putatively corresponded to a dimer of caffeoyl quinic acid. In particular, in the TOF-MS/MS spectrum of metabolite 3, whose isomer, with antimicrobial activity against *Staphylococcus aureus* and *Escherichia coli*, was isolated from *Hymenocrater calycinus* (Boiss.) Benth. [32], the deprotonated molecular ion at *m*/*z* 371.1052 underwent neutral loss of 18 Da, providing the less intense ion at *m*/*z* 353.0872 (caffeoyl quinate), and gave rise to the abundant ion at *m*/*z* 191.0554 (quinate) (Figure 2F). Deprotonated molecular ion of metabolite **12**, at *m*/*z* 707.1845, underwent neutral loss of 174 Da and 192 Da, whose presence was further confirmed by fragment ion at *m*/*z* 191.0557. Moreover, according to TOF-MS/MS spectrum, the ring arrangement could be of the β-truxillic acid type; cleavages along the two axes of central core provided the fragment ions at *m*/*z* 463.1085, 353.0870 and 243.0651 (Figure S1).

Furthermore, coumaroyl derivatives were also identified: compounds **11** and **20**, at *m*/*z* 337.0921(18), were tentatively identified as 3-*O*- and 5-*O*-*p*-coumaroyl quinic acid (*p*CoQAs) in accordance with the molecular formula C_16_H_18_O_9_ (Figure 2, panels G and H), whereas compound **14**, showing the [M−H]^−^ ion at *m*/*z* 325.0921, was tentatively identified as *p*-coumaroyl hexoside (Figure 2I). In all TOF-MS/MS spectra, the fragment ion at *m*/*z* 163.04 was detected, highlighting the presence of deprotonated coumaric acid (C_9_H_8_O_3_). Compounds **21** and **22**, whose deprotonated molecular ion was in accordance with the molecular formula C_16_H_16_O_8_, could be caffeoylshikimic acids (CSAs) [31]. Both metabolites showed fragment ions at *m*/*z* 179.03 and 161.02, generated by neutral loss of 156 Da (shikimic acid-H_2_O) and 174 Da (shikimic acid) (Figure 2, panels J and K). The intensity of this latter allowed us to identify compound **22** as 4-*O*-CSA. 

#### 3.1.2. Flavonoids 

**Figure 3:**
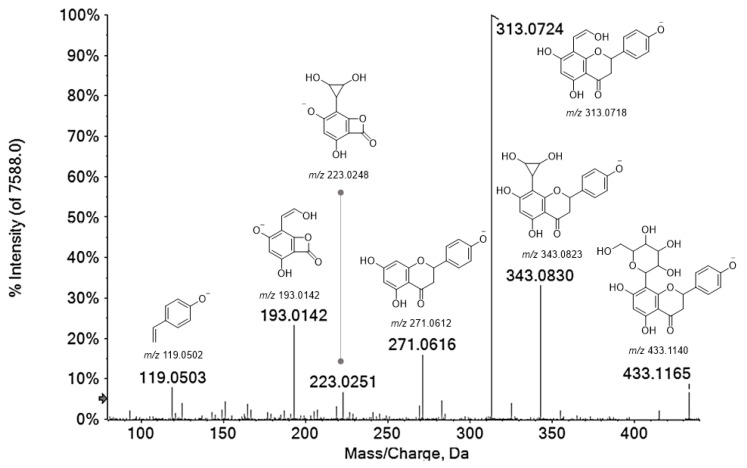
TOF-MS^2^ spectrum of metabolite **24**. The chemical structure of each product ion is depicted, and theoretical *m*/*z* ratio is indicated below.

Compounds **18, 24, 25, 34** were tentatively identified as flavanones. Compound **18** with the [M−H]^−^ ion at *m*/*z* 449.1097 was tentatively identified as eriodictyol-7-*O*-hexoside. In fact, the [M−H]^−^ ion provided in the TOF-MS/MS experiment an abundant deprotonated aglycone ion (33.9%) at *m*/*z* 287.0553, following the neutral loss of a hexose moiety, and the fragment ion at *m*/*z* 259.0609 (base peak) by neutral loss of CO (28 Da) and hexose moiety (162.05 Da). Metabolites **24, 25** and **34** were hypothesized to be *C*-glycosylated flavanones, which are much less studied than *O*-glycosides, but endowed with several health benefits, such as antioxidant, anticancer, antitumor and anti-diabetic activities [33]. The occurrence of naringenin-*C*-glycosides was previously described by Hoffman et al. [25], but though an HPLC-MS/MS via Multiple Reaction Monitoring (MRM) analysis was performed, these authors did not provide sufficient MS/MS details to prove the presence of these compounds in beech leaves. The almost superimposable MS/MS fragmentation patterns were in accordance with naringenin *C*-hexoside isomers, likely bearing different sugar moieties that reasonably explained the different retention times (Figure 3). In fact, the deprotonated molecular ion at *m*/*z* 433.11 provided the ions [Ag+71]^−^ at *m*/*z* 343.08 and [Ag+41]^−^ at *m*/*z* 313.07 (base peak) by the ^0,2^X and ^0,3^X cross-ring cleavage of hexoside, likely linked at C-8 position. This hypothesis was supported by very low intensity of dehydrated fragment ions at *m*/*z* 415.10 and 325.07, which are known to be much more pronounced for 6-*C* isomers [34]. Although the occurrence of this kind of compounds is quite unusual, some literature data consoled our hypothesis. Indeed, naringenin 8-*C*-β-glucopyranoside (isohemiphloin) was isolated from *Eucalyptus hemiphloia* F. Muell. (Myrtaceae), and its 6-*C* isomer (hemiphloin) was identified also in *Ononis vaginalis* M.Vahl. (Fabaceae), *Tulipa gesneriana* L. and *Ulmus wallichiana* Planch., beside eriodictyol 6-*C*-β-d-glucopyranoside [35]. 

**Figure 4:**
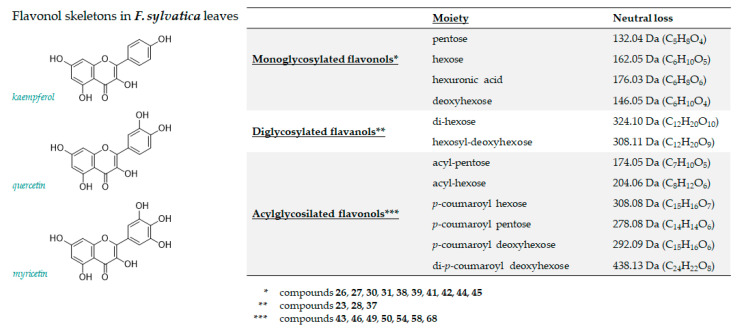
Chemical structures of major flavonol aglycones tentatively identified in the extract under study. Neutral losses with related molecular formulas have been reported to summarize and schematize the different moieties attached to aglycones.

Different flavan-3-ols mono- and diglycosides have been identified too. In Figure 4, neutral losses and related molecular formulas of their main fragmentations are schematized. In particular, considering flavonols group, monoglycosides were 82.2%, diglycosides account 17.2%, and (acyl)-glycoside flavonols were only 0.51%. The deprotonated compounds **26** (*m*/*z* 479.0844) and **27** (*m*/*z* 449.0746) were putatively identified as myricetin 3-*O*-hexoside and myricetin 3-*O*-pentoside, respectively. In fact, the loss of 162 Da (hexose moiety) and 132 Da (pentose moiety) provided in both cases the fragment ion at *m*/*z* 317.02, attributable to myricetin, together with its aglycone radical anion at *m*/*z* 316.02, whose abundance allowed us to hypothesize the C-3 linkage of sugars moieties. 

Compounds **30, 31** and **38** shared quercetin as aglycone and they were identified as quercetin 3-*O*-hexoside (**30**), quercetin 3-*O*-hexuronide (**31**), and quercetin 3-*O*-pentoside (**38**), respectively [3,11,31]. Kaempferol glycosides appeared to be more abundant. Among them, metabolites **39** and **41** were putatively identified as 3-*O*-galactopyranoside and 3-*O*-glucopyranoside derivatives. These latter were more abundant among kaempferol derivatives, representing the 36.2%. The shorter retention time of the 3-*O*-galactopyranoside derivative [36], the natural higher abundance of the 3-*O*-glucopyranoside isomer, and the relative intensity of fragment ion at *m*/*z* 285 allowed us to discriminate the two isomers. The mass spectrum of compound **42** was in accordance with kaempferol 3-*O*-pentoside. In fact, MS/MS spectrum of the deprotonated molecular ion at *m*/*z* 417.0844 generated, by neutral loss of 132 Da (dehydrated pentose moiety), the [M−H-C_5_H_9_O_4_]^−^ ion at *m*/*z* 285.0410, whose corresponding radical aglycone ion at *m*/*z* 284.0337 was the peak base. Instead, the neutral loss of 204.06 Da allowed us to tentatively identify metabolite **43** (at *m*/*z* 489.1054) as kaempferol (acetyl)-hexoside. Analogously, compound **46** at *m*/*z* 459.0938 was putatively identified as kaempferol (acetyl)-pentoside, whose glycosidic bond cleavage (−174.05 Da) provided a low aglycone ion and an abundant radical aglycone ion. Finally, the different [aglycone-H]^−^/[aglycone-H]^●−^ (*m*/*z* 285.0407/284.0328) ratio in the TOF-MS^2^ spectrum of compound **45** was in accordance with a 7-*O*-deoxyhexosyl kaempferol, whereas, among flavanol diglycosides, compounds **23** and **28**, sharing the [M−H]^−^ ion at *m*/*z* 609.1466(81) and the same molecular formula (C_27_H_30_O_16_), were tentatively identified as kaempferol dihexosides. 

Compound **37** showed a TOF-MS/MS spectrum in accordance with isorhamnetin hexosyl deoxyhexoside. In fact, the break of 3-*O*-glycosidic cleavage, with a loss of 308.11 Da (hexose+deoxyhexose), gave rise to the [aglycone-H]^●−^ and [aglycone-H]^−^ ions at *m*/*z* 314.0423 and 315.0501, respectively. A further loss of 15 Da, providing fragment ions at *m*/*z* 299.0176, supported the structural hypothesis. Metabolites **49, 50** and **68** were further identified as *p*-coumaroyl derivatives of kaempferol hexoside (**49** and **50**) and deoxyhexoside (**68**). The [M−H]^−^ ion at *m*/*z* 593.1326(31) of the compounds **49** and **50** led to [aglycone-H]^−^ and [aglycone-H]^●−^ ions by neutral loss of 308.0951 Da and 309.1027 Da, in accordance with a *p*-coumaroylhexosyl moiety. In the TOF-MS/MS spectra the neutral loss of 146.04 Da, in accordance with the dehydrated *p*-coumaric acid, provided the ion at *m*/*z* 447.0954(8), whose intensity allowed us to hypothesize the linkage of the hydroxycinnamic acid to the saccharide moiety. Instead, metabolite **68**, tentatively identified as kaempferol di-*p*-coumaroyl deoxyhexoside, showed the [M−H]^−^ ion at *m*/*z* 723.1752 that dissociated giving fragment ions at *m*/*z* 577.1391 and 559.1232, according to neutral loss of dehydrated (or not) *p*-coumaric acid. The fragment ion at *m*/*z* 437.1261 (48.6%) consisted in the acylated sugar moiety (di-*p*-coumaroyl-deoxyhexoside-H_2_O), according to the observed neutral loss of 438.13 Da from deprotonated molecular ion that, through heterolytic or homolytic cleavage, provided the ions at *m*/*z* 285.0404 and 284.0322. The deprotonated molecular ion for compound **58** (at *m*/*z* 577.1378) was in accordance with a flavone acylglycoside, likely luteolin *p*-coumaroyl-deoxyhexoside. In the TOF-MS/MS spectrum, the [M−H]^−^ ion dissociated supplying the abundant fragment ion [aglycone-H]^−^ at *m*/*z* 285.0409, through the neutral loss of 292.0975 Da (*p*-coumaric acid+deoxyhexose). The absence of fragment ions at *m*/*z* 255 and *m*/*z* 227, which allowed flavonols to be distinguished from flavones, allowed us to identify the aglycone as luteolin. 

The fragmentation pattern of compound **13** was in accordance with the presence of procyanidin (B-type). In fact, the [M−H]^−^ ion at *m*/*z* 577.1355 dissociated losing the A-ring of the flavanolic unit (−126 Da) through the heterocyclic ring fission (HRF) mechanism, producing the fragment ion at *m*/*z* 451.1030. The fragment ion at *m*/*z* 425.0863 was due to retro-Diels Alder (RDA) mechanism, and the monomeric unit at *m*/*z* 289.0710 was generated through the quinone methide fission [37]. Moreover, the deprotonated molecular ion at *m*/*z* 289.0708 for compound **15** was in accordance with the molecular formula C_15_H_14_O_6_ and (epi)catechin compound. The decarboxylation (−44 Da) and subsequent loss of an ethenone unit (C_2_H_2_O), generated fragment ions at *m*/*z* 245.0818 and 203.0709, respectively; from the latter, the loss of lateral chain as 2-methylene-2H-pyran (C_6_H_6_O; −94.04 Da) for nucleophilic attack of hydroxyl group to benzylic carbon, provided base peak at *m*/*z* 109.0290. Following the benzofuran-forming fission reaction from deprotonated molecular ion the ion at *m*/*z* 123.0447 was formed [37]. 

#### 3.1.3. Lignans

Compounds **29, 32, 36**, and **47** were tentatively identified as lignans. In particular, metabolite **29** was tentatively identified as isolariciresinol hexoside, whose deprotonated molecular ion at *m*/*z* 521.2046 underwent neutral loss of sugar moiety providing the fragment ion at *m*/*z* 359.1504, from which the loss of formaldehyde provided the abundant ion at *m*/*z* 329.1396. Compounds **32** and **36** could be neolignan-*O*-deoxyhexoside isomers; these metabolites were previously reported as unidentified compounds [11]. Instead, two neolignan-9′-*O*-deoxyhexoside stereoisomers were isolated and identified by NMR in *Fagus hayatae* Palib. ex Hayata lea [38]. This finding is in line with the occurrence of these metabolites, which were also isolated in *Pinus thunbergii* [39]. Beside fragment ions at *m*/*z* 491.1926(61) and 473.1824(30), which are derived from dehydration reactions, the bond cleavage between the two phenylpropanoid units provided fragment ions at *m*/*z* 179.0712(13) (base peak) and 313.1290(99). In Figure S2 TOF-MS/MS spectra were reported, and the hypothesized chemical structures for the most abundant fragment ions were provided.

The metabolite **47**, whose tentative characterization was possible with a slight modification of Q-TOF parameters (Figure S3), was identified as 4,9,9′-trihydroxy-3,3′,5′-8-*O*-4′-neolignan-7-*O*-deoxyhexoside [40]. The loss of 212.06 Da, likely corresponding to trimethoxygallic acid, generated the fragment ion at *m*/*z* 343.1410, which in turn lost a methyl radical to generate the ion at *m*/*z* 328.1177. The presence of the deoxyhexose moiety was confirmed by loss of 146.06 Da, providing the ion at *m*/*z* 197.0821. As for compounds **32** and **36**, the fragment ion derived by sugar loss has a very low intensity, likely justified by an intramolecular hydrogen bond. Compound **48** with the [M−H]^−^ ion at *m*/*z* 551.2167, was supposed to be a lignan (Figure S4): 9′-hydroxy-7′-propen-3′,5′-dimethoxyphenyl-3-methoxyphenyl-7,9-propanediol-4-*O*-hexoside [41]. The fragment ion at *m*/*z* 209.0815 was attributable to a sinapyl alcohol moiety, from which ions at *m*/*z* 194.0579 and 179.0344 were generated. To the best of our knowledge, this lignan has never been identified in *F. sylvatica*.

Flavolignan compounds, such as compounds **33** and **35** (at *m*/*z* 451.1047(46)) were tentatively identified. These compounds were likely cinchonain-I isomers, which are phenylpropanoid-substituted catechin, characterized by the ion at *m*/*z* 341.0669(74), generated from the loss of the 3,4-dihydroxyphenyl group (−110 Da). Subsequently, through the HRF mechanism and the RDA mechanism, the ions at *m*/*z* 189.0186(78) and 177.0185(93) were formed, respectively.

#### 3.1.4. Fatty Acids

The investigated extract also showed the occurrence of mono- or poly-hydroxylated fatty acids, whose presence, together with epoxy derivatives, was previously identified by Matzke et al. [42]. In particular, metabolite **52**, was tentatively identified as trihydroxy octadecadienoic acid (*m*/*z* 327.2185). It underwent dehydration processes, providing fragment ions at *m*/*z* 309.2087 and 291.1962. The C12-C13 bond cleavage provided the ion at *m*/*z* 229.1448, which in turn lost 58 Da (C_3_H_6_O) and 46 Da, to generate fragment ions at *m*/*z* 183.1385 and 171.1026, respectively (Figure S5). 

Compound **56** was putatively identified as hydroxyhexadecanoic acid, e.g., 16-OH-hexadecanoic acid [33]), whose dehydration provided a low intensity ion at *m*/*z* 269.2114, whereas TOF-MS/MS spectrum of compound **61** indicated it could be dihydroxyoctadecedienoyl quinic acid. The loss of dehydrated quinic acid, identified by fragment ion at *m*/*z* 191.0559, produced an abundant ion at *m*/*z* 311.2230 that corresponded to the fatty acid. In addition to fragment ions at *m*/*z* 293.2117 and 275.2012, obtained by losses of hydroxy groups, also a fragment ion at *m*/*z* 223.1703, to cleavage between C-4 and C-5, was detected (Figure S6). 

Compound **63** was putatively a hydroxyoctadecatrienoic acid, maybe with hydroxyl group at carbon C-10. In fact, the β-scission of the alcoholic group provided the ion [M−H-110]^−^ at *m*/*z* 183.1385 identified as base peak. An allyl scission provided ions at *m*/*z* 221.1539 and 211.1338 from dehydrated ion (*m*/*z* 275.2014) and deprotonated molecular ion, respectively. Compound **65** showed TOF-MS/MS spectrum in accordance with 15,16-dihydroxy-9,12-octadienoic acid [43]. Product ions deriving from water losses were detected at *m*/*z* 293.2125 and 275.2017. Furthermore, allyl scission gave a low abundance ion at *m*/*z* 253.1804, whereas the β-fission of the alcoholic hydroxyl group provided an abundant ion at *m*/*z* 223.1707. Compound 69 (at *m*/*z* 675.3629) was putatively identified as a linolenic acid glyceryl-dihexoside. In fact, the product ion at *m*/*z* 277.2158 corresponded to the fatty acid moiety, whereas glyceryl dihexoside could be identified by fragment ions at *m*/*z* 415.1454 and 397.1344; from the latter the loss of 92 Da, identifying a glycerol unit, provided the ion at *m*/*z* 305.0848. In Figure S7, the TOF-MS/MS spectrum and a putative fragmentation pathway are reported.

#### 3.1.5. Other Minor Compounds

None of the remaining compounds were assignable to any of the previously discussed classes. Briefly, compound **1** was likely quinic acid, whereas metabolite **2** was putatively identified as tyrosine hexoside, based on the presence of the ion at *m*/*z* 180.0664 (deprotonated tyrosine), formed after the neutral loss of the hexose moiety. Metabolite **19** with the [M−H]^−^ ion at *m*/*z* 387.1666 was tentatively identified as 12-hydroxyjasmonate (tuberonic acid) [18]. 

The TOF-MS/MS spectrum of compound **40** showed fragment ions at *m*/*z* 125.0237, 101.0240 and 99.0451 in accordance with a 3-hydroxy-3-methylglutaryl substitution of hexenyl hexoside [44]. Thus, the metabolite was putatively a 3-hydroxy-3-methylglutaryl-(*O*-hexenyl) hexoside, from which the loss of 144 Da (HMG) gave the fragment ion at *m*/*z* 261.1311. Metabolites **59** and **60,** with a [M−H]^−^ ion at *m*/*z* 955.4948(41), were tentatively identified as triterpenoid saponins with oleanolic acid as aglycone, previously characterized in *F. sylvatica* leaves through spectroscopic techniques [13], but never by mass spectrometry analysis. In order to obtain useful information about the fragmentation pattern, declustering potential and collision energy values were optimized; thus, metabolite **59** was tentatively identified as 28-(D-glucopyranosyloxy)-28-oxoolean-12-en-3β-yl-3-O-(β-D-glu-copyranosy1)-β-D-glucopyranosiduronic acid. The deprotonated molecular ion lost the hexosyl moiety, providing the ion at *m*/*z* 793.4368(69), detected in TOF-MS/MS spectra of both metabolites, and underwent ^0,2^X cross-ring cleavage of hexuronic acid ring, to give the ion at *m*/*z* 659.4152, diagnostic for the identification of metabolite **60** [13,45] (Figure S8). The neutral loss of 176 Da (dehydrated hexuronic acid) from ion at *m*/*z* 631.38 provided the ion at *m*/*z* 455.3505(16), most likely oleanolic acid. 

### 3.2. Relative Quantitation of F. sylvatica Leaf Chemical Constituents

**Figure 5:**
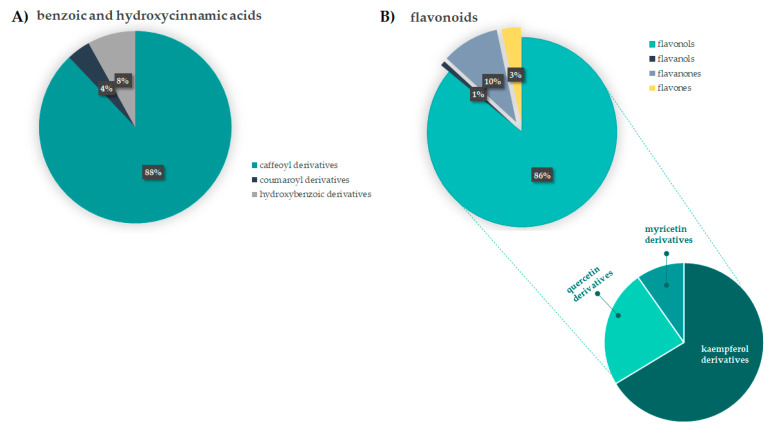
Semi-quantitative representation of beech leaf extract constituents. (**A**) Relative content of coumaroyl, caffeoyl and hydroxybenzoic derivatives into the class of benzoic and hydroxycinnamic acids; (**B**) Relative content of different flavonoid subfamilies.

Hydroxycinnamic acid (HCA) derivatives constitute a considerable part of low molecular weight phenol compounds. Caffeoyl-based HCA compounds were the most abundant, as they account for 88.0% of the compounds with C_6_C_1_ and C_6_C_3_ carbon skeleton. In particular, chlorogenic acids, such as 3-*O*- and 5-*O*-caffeoyl quinic acid, were 27.9% and 54.9%, respectively, whereas coumaroyl derivatives were less represented (~4.0%). The interest in chlorogenic acids is due to the plethora of beneficial effect ascribed to these substances, for which certain fruits, vegetables, spices are the main dietary sources. In particular, 5-*O*-caffeoyl quinic acid, firstly analyzed for its antioxidant, anti-inflammatory, and antitumor activity [46] was found to play multiple and key roles in protecting humans at neuronal, cardiovascular, and gastrointestinal levels. It was also implied in glucose and lipid metabolic regulation [47]. Besides caffeoyl derivatives, flavonoids appeared to be in appreciable amount. Flavonols were the main constituents of this class, with a relative percentage of 86.0%. Figure 5 shows the relative content of benzoic/hydroxycinnamic acids class, and flavonoids.

Kaempferol derivatives were the most abundant (66.0%), followed by quercetin derivatives (24.0%), and myricetin derivatives (10%). The abundance of flavonoids in different organs of *F. sylvatica*, such as leaves or bark [12,48,49], was stated by different literature data, in which kaempferol, quercetin, myricetin, luteolin or naringenin derivatives were identified. Unusual naringenin-*C*-glycosides are as part of flavanone class, which is commonly associated to different benefits due to their ability to act as free radical-scavenger. Juice and peel of citrus fruits are the main dietary sources of these compounds [50], which were also clinically evaluated for cardiovascular disease protection. The anti-inflammatory, antioxidant and soothing effects of flavonoids are broadly exploited for food, drugs or cosmetics production [51]. 

Apart from antiradical and reducing activities, food-derived flavonoids were shown to prevent non-communicable diseases on-set, and to exert pro-oxidant effect in cancer cells, thus increasing ROS levels and apoptosis rate. Several epidemiological evidences suggest that kaempferol-rich foods reduce the risk of liver, colon and skin cancer [52], whereas the uncountable properties of quercetin and its derivatives give rise recently-filed patents for disparate therapeutic applications [53]. Towards a green and sustainable waste valorisation chain, the employment of *F. sylvatica* leaves should be pursued. 

### 3.3. Beech Leaf Alcoholic Extract Showed Antioxidant Efficacy in Cell-Free Assays

**Figure 6:**
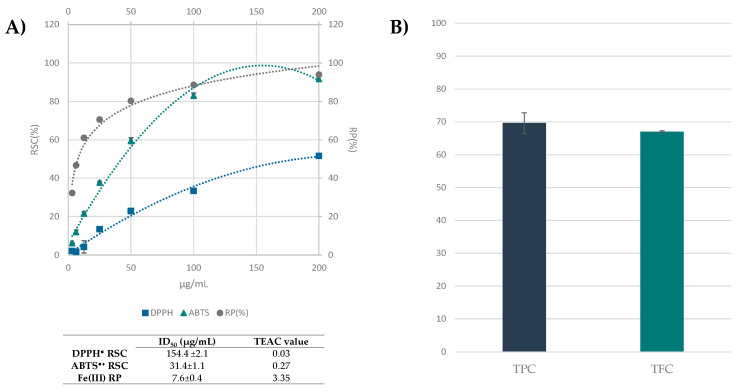
(**A**) Radical scavenging activity (RSC%) of *F. sylvatica* leaf extract vs. 2,2-diphenyl-1-picrylhydrazy (DPPH) radical (■) and 2,2′-azino-bis(3-ethylbenzothiazoline-6-sulfonic acid (ABTS) radical cation (▲). Fe(III) Reducing Power (RP%) is also measured (●). ID_50_ values (µg/mL) and TEAC (Trolox Equivalent Antioxidant Capacity) values are tabulated. Values reported are the mean ± SD of three independent measurements. (**B**) Total Phenol Content (TPC) were expressed as mg of gallic acid equivalent per g of extract, and Total Flavonoid Content (TFC) were expressed as mg of quercetin equivalent per g of extract. Values reported are the mean ± SD of three independent measurements.

The alcoholic leaf extract was evaluated for its antioxidant capability by means of assays, involving stable radicals, such as DPPH^●^ and ABTS^●+^ tests, and by ferricyanide FRAP assay. Folin-Ciocalteu test, which employs phospho-tungsto-molybdate, was also performed, together with the colorimetric determination of total flavonoid content. Data acquired showed that the extract was effective in scavenging ABTS^●+^ with an ID_50_ and TEAC value equal to 31.4 ± 1.10 µg/mL and 0.27, respectively, and it was able to markedly reduce ferric ions, also at the lowest tested dose (Figure 6A). The total phenol content (TPC) was 69.64 ± 3.1 mg of gallic acid equivalents per g of extract (Figure 6B). Tanase et al. [24], who investigated TPC in beech bark hydroalcoholic extract, found it was 76.49 mg GAE/g plant material. Comparably, bark extracts of *F. sylvatica*, obtained by means of different extractive methods were screened for their antiradical activity and phenol content, and their diversity in catechins, taxifolin glycosides, procyanidins, syringic acid or coniferyl alcohol glycosides were further estimated by chromatographic techniques hyphenated to mass spectrometry [10,24,48,54]. Among the few studies on beech leaf, a comparative qualitative study was carried out on hydroalcoholic extracts (EtOH:water, 7:3, *v*/*v*) of beech leaves collected in Romania; it was evidenced that TPC significantly varied based on leaf harvest times [12,55], reaching the maximum TPC value (33.55 mg/g GAE), when leaves were collected in September. According to UHPLC-ESI-Q*q*TOF analysis, a high content of flavonoid compounds was spectrophotometrically determined (Figure 6B).

### 3.4. Beech Leaf Alcoholic Extract Decreased Intracellular ROS in Caco-2 Cells

**Figure 7:**
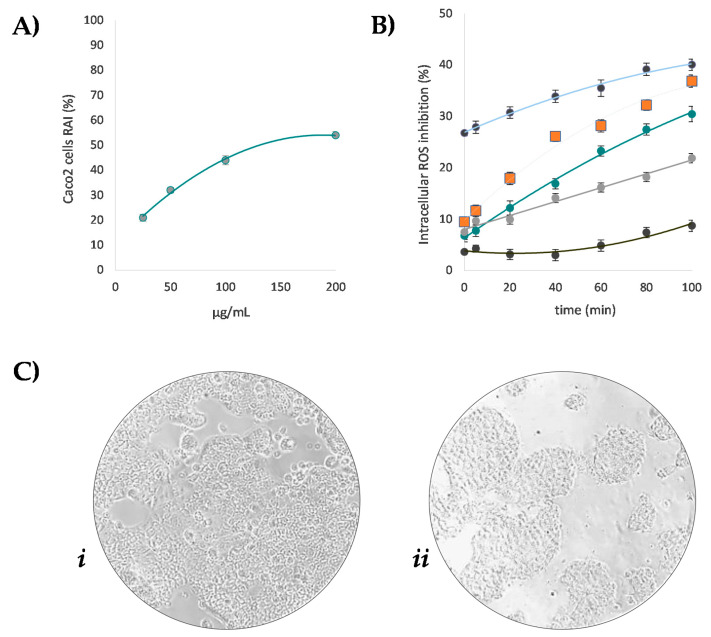
(**A**) Redox activity inhibition (RAI%) of *F. sylvatica* leaf extract towards Caco-2 cells. (**B**) ROS intracellular inhibition (%) of *F. sylvatica* leaf extract ● 200 μg/mL, ● 100 μg/mL, ● 50 μg/mL ● 25 μg/mL, and of quercetin 10 μM (■). Values reported are the mean ± SD of three independent measurements. (**C**) Representative images of untreated Caco-2 cells (***i***) and treated Caco-2 cells (***ii***) acquired by Inverted Phase Contrast Brightfield Zeiss Primo Vert Microscope.

The extract richness in polyphenols and the positive responses of cell-free antioxidant tests paved the way to the evaluation of its antioxidant potential in cell-based assays. To this purpose, Caco-2 cell line represents a useful model, able to predict in vivo behavior. In fact, besides being widely used as an in vitro model for the intestinal epithelial barrier, they were reported to offer a distinctive advantage in screening intestinal absorption of natural antioxidants after oral intake [21]. Thus, with the aim to exploit beech leaf phytochemicals in the formulation of nutraceuticals, the ability of the extract to counteract reactive oxygen species (ROS) overproduction was preliminarily evaluated in Caco-2 cells [49]. In fact, metabolic activity is strongly related to ROS production, which intracellularly originate in the mitochondrial respiratory chain, also producing toxic metabolic byproducts [56]. Thus, redox mitochondrial activity was first assessed by means of MTT test (Figure 7A). The beech leaf alcoholic extract exhibited dose-response inhibition of the metabolic activity of Caco-2 cells, with an ID_50_ equal to 148.4 μg/mL. In the cell model, after co-exposing Caco-2 cell monolayer to increasing dose levels of the extract and to the fluorescent probe DCFH-DA, oxidative stress is induced by 2,2-azobis(2-amidinopropane) dihydrochloride (AAPH). Based on fluorescence levels, which are related to the oxidation degree, it was observed that the extract markedly reduced the cellular fluorescence compared to the untreated cells, when it was tested at dose level equal to 200 μg/mL (Figure 7B). The other doses tested, while exhibiting less pronounced efficacy, showed a strongly time-dependent activity. Overall, the dose-dependent capability of the beech leaf extract was ascertained. Considering the cell basal rate in oxidizing species, the outcome of the investigated beech on redox balance could be due to extract-induced mitochondrial alterations. The effect was compared to that exerted by pure standard quercetin, which was tested at 10 μM, based on cell viability data and according to literature [21,57]. In fact, the peroxyl radical scavenging activity of quercetin in Caco-2 cells was broadly proved [21], and it was found that doses of the flavonol higher than 20 μM markedly affected colon cells viability [57]

## 4. Conclusions

*Fagus sylvatica* leaves, investigated through an untargeted UHPLC-HR-MS/MS analysis, revealed their richness in flavonoids, mainly flavonol monoglycosides. Thus, the recovery of this renewable plant source could favor an application to create beech leaf-derived products, in the form of food or dietary supplements or herbal remedies for human or animal health. Cell-free assays evaluating the antiradical and reducing capability, as well as preliminary antioxidant capability in Caco-2 cells, strengthened this exploitation hypothesis. Thus, the promising antioxidant results herein reported could be the basis for the further phytochemical investigation of *F. sylvatica*, and the use of different extractive and chromatographic approaches aimed at recovering high amounts of pure beech bioactive compounds.

## Figures and Tables

**Table 1:** TOF-MS and TOF-MS/MS data of compounds tentatively identified in *F. sylvatica* L. methanolic leaf extract. Compounds are numbered based on their retention time (RT) in the total ion current chromatogram (RDB = Ring Double Bond equivalent value).

Peak	RT (Min)	Tentative Assignment	Formula	[M−H]^−^ Calc. (*m*/*z*)	[M−H]^−^ Found (*m*/*z*)	Error (ppm)	RDB	MS/MS Fragment Ions (*m*/*z*) and Relative Intensity
Benzoic and hydroxycinnamic acid derivatives								
3	0.699	Hydroxycaffeoyl quinic acid	C_16_H_20_O_10_	371.0984	371.0970	−3.7	7	371.1052(3.4); 353.0872(3.4); 341.0900(3.4); 191.0554(100); 173.0467(5.3); 135.0447(5.3)
4	0.875	Dihydroxybenzoic acid hexoside	C_13_H_16_O_9_	315.0719	315.0722	−0.8	6.0	315.0714(18.8); 153.0191(16.0); 152.0112(60.5); 109.0294(34.8); 108.0217(100)
5	1.034	Hydroxybenzoic acid hexoside	C_13_H_16_0_8_	299.0772	299.0769	−1.1	6	299.0780(29.3); 239.0572(17.8); 179.0341(41.4); 137.0242(58.6); 136.0159(17.8); 121.0290(100); 93.0345 (41.4)
6	1.073	Dihydroxybenzoic acid	C_7_H_16_O_4_	153.0193	153.0198	3.1	5	109.0291(100); 108.0215(87.5); 91.0185(12.4); 81.0341(8.7)
7	1.226	3-*O*-Caffeoyl quinic acid	C_16_H_18_O_9_	353.0878	353.0893	0.8	8	191.0565(100); 179.0354(48.1); 135.0453(44.5); 134.0370(5.8)
8	1.495	Caffeoyl acid hexoside	C_15_H_18_O_9_	341.0874	341.0878	−1.2	7	341.0886(9.4); 179.0339(22.0); 161.0244(100); 135.0444(9.4); 89.0243(18.6)
9	1.514	Caffeoyl threonic acid	C_13_H_14_O_8_	297.0616	297.0613	−0.1	7	179.0344(9.6); 135.0304 (100); 117.0192 (6.6); 89.0246(11.6)
11	1.921	3-*O*-*p*-Coumaroyl quinic acid	C_16_H_18_O_8_	337.0929	337.0921	−2.3	8	191.0550(61.4);173.0493(4.5);163.0396(100); 119.0946(74.6)
12	1.921	Caffeoyl quinic acid dimer	C_32_H_36_O_18_	707.1829	707.1831	0.3	15	707.1845(100); 533.1307(2.3); 515.1192(3.8); 463.1085(3.5); 353.0870(8.2); 323.0546(3.2); 243.0651(2.6); 191.0557(32.1)
14	2.058	*p*-Coumaroyl acid hexoside	C_15_H_18_O_8_	325.0929	325.0921	−2.4	7	163.0394(46.2); 119.0499(100)
16	2.370	5-*O*-Caffeoyl quinic acid	C_16_H_18_0_9_	353.0878	353.0878	0	8	191.0562(100); 85.0296(3.4)
17	3.048	Caffeoyl propionic acid	C_12_H_14_O_6_	253.0718	253.0718	0.1	6	253.0726(19.0); 179.0347(19.0); 161.0241(100); 135.0454(63.4); 133.0295(70.8)
20	3.576	5-*O*-*p*-Coumaroyl quinic acid	C_16_H_18_O_8_	337.0929	337.0918	−3.2	8	191.0560(100); 173.0442(4.0); 163.0405(6.0); 119.0491(6.7); 93.0346(16.9); 87.0073(3.7); 85.0290(2.7)
21	3.929	5-*O*-Caffeoyl shikimic acid	C_16_H_16_O_8_	335.0772	335.0773	−0.4	9	335.0755(3.9); 179.0346(61.5); 173.0454(2.3); 161.0239(24.5); 135.0446(100); 134.0372(6.9); 93.0344(6.9)
22	4.047	4-*O*-Caffeoyl shikimic acid	C_16_H_16_O_8_	335.0772	355.0768	−1.3	9	335.0748(3.9); 179.0350(21.5); 161.0237 (100); 135.0449 (22.7)
Flavonoids								
13	1.921	Procyanidin (B *type*)	C_30_H_26_O_12_	577.1352	577.1355	0.6	18	577.1366(22.0); 451.1030(14.9); 425.0863(27.3); 407.0772(100); 381.0998(12.6); 299.0523(11.1); 289.0710(79.4); 245.0444(18.5); 125.0240(79.4)
15	2.136	Catechin	C_15_H_14_O_6_	289.0718	289.0708	−3.3	9	289.0696(38.3); 245.0818(30.8); 221.0797(30.8); 205.0505(15.0); 203.0709(54.1); 187.0381(30.8); 179.0365(23.3); 151.0403(40.6); 137.0246(30.8); 125.0238(30.8); 123.0447(84.2); 109.0290(100)
18	3.048	Eriodictyol 7-*O*-hexoside	C_21_H_22_O_11_	449.1089	449.1097	1.7	11	449.1097(2.5); 421.1142(8.5); 313.0717(2.5); 301.0709(9.4); 287.0553(33.9); 259.0609(100); 243.0661(8.2)
23	4.125	Kaempferol 3,7 di-*O*-hexoside	C_27_H_30_O_16_	609.1461	609.1466	0.8	13	609.1517(100); 489.1064(25.2); 447.0941(82.9); 446.0886(49.6); 285.0390(82.9); 284.0300(25.2); 283.0241(28.1)
24	4.637	Naringenin 8-*C*-hexoside (1)	C_21_H_22_O_10_	433.1140	433.1145	1.1	11	433.1165(7.1); 343.0830(34.3); 313.0724(100); 271.0616(15.9); 223.0251(6.7); 193.0142(24.0); 119.0503(5.4)
25	4.917	Naringenin 8-*C*-hexoside (2)	C_21_H_22_O_10_	433.1140	433.1145	1.1	11	433.1146(6.2); 343.0819(30.6); 313.0714(100); 271.0603(14.8); 223.0243(5.6); 193.0136(24.3); 165.0817(4.8); 119.0501(9.1)
26	5.149	Myricetin 3-*O*-hexoside	C_21_H_20_O_13_	479.0831	479.0844	12	2.7	479.0855(32.8); 317.0300(14.7); 316.0226(100); 287.0193(6.1); 271.0239(11.1)
27	5.772	Myricetin 3-*O*-pentoside	C_20_H_18_O_12_	449.0725	449.0746	4.6	12	449.0728(26.4); 317.0287(7.5); 316.0216(100); 287.0183(10.5); 271.0241(18.4);
28	5.812	Kaempferol 3-*O*-dihexoside	C_27_H_30_O_16_	609.1461	609.1481	3.3	13	609.1481(28.6); 285.0408(69.3); 284.0320(100); 255.0289(7.3)
30	6.046	Quercetin 3-*O*-hexoside	C_21_H_20_O_12_	463.0882	463.0893	2.4	12	463.0902(21.1); 301.0352(60.3); 300.0274(100); 271.0246(28.6); 255.0292(15.9)
31	6.223	Quercetin 3-*O*-hexuronide	C_21_H_18_O_13_	477.0675	477.0692	3.6	13	477.0694(5.5); 301.0354(100); 283.0237(2.5); 255.0295(2.3); 178.9974(6.3); 151.0029(6.2)
34	6.722	Naringenin 8-*C*-hexoside (3)	C_21_H_22_O_10_	433.1140	433.1154	3.2	11	433.1144(3.1); 343.0822(34.7); 313.0715(100); 271.0600(19.4); 269.0818(3.9); 223.0235(7.0); 205.0127(3.0); 193.0141(31.8); 151.0039(7.1); 119.0501(10.8)
37	7.024	Isorhamnetin hexosyl deoxyhexoside	C_28_H_32_O_16_	623.1618	623.1642	3.9	13	623.1636(100); 315.0501(11.1); 314.0423(70.7); 299.0176(15.2); 285.0423(5.4); 271.0256(5.4)
38	7.196	Quercetin 3-*O*-pentoside	C_20_H_18_O_11_	433.0776	433.0795	4.3	12	433.0796(16.5); 301.0355(19.2); 300.0281(100); 271.0247(21.7); 255.0295(10.4)
39	7.240	Kaempferol 3-*O*-galactopyranoside	C_21_H_20_O_11_	447.0933	447.0946	2.9	12	447.0944(39.4); 327.0500(2.6); 285.0397(27.2); 284.0322(100); 255.0293(40.7); 227.0342(23.5)
41	8.003	Kaempferol 3-*O*-glucopyranoside	C_21_H_20_O_11_	447.0933	447.0943	2.3	12	447.0943(35.5); 285.0398(55.2); 284.0322(100); 255.0295(47.0); 227.0345(27.7)
42	8.897	Kaempferol 3-*O*-pentoside	C_20_H_18_0_10_	417.0827	417.0844	4.0	12	417.0858(31.8); 285.0410(22.1); 284.0337(100); 255.0306(53.1); 227.0355(31.5)
43	9.203	Kaempferol (acetyl)-hexoside	C_23_H_22_O_12_	489.1039	489.1054	3.2	13	489.1071(36.3); 285.0383(37.7);284.0313(100); 255.0307(28.4); 227.0329(12.3)
44	10.260	Kaempferol 7-*O*-pentoside	C_20_H_18_0_10_	417.0827	417.0833	3.3	12	417.0833(38.1); 285.0398(100); 284.0330(85.6); 255.0301(52.1); 227.0355(38.1)
45	11.672	Kaempferol 7-*O*-deoxyhexoside	C_21_H_20_O_10_	431.0984	431.0999	3.5	12	431.1006(24.2); 285.0407(100); 284.0328(87.5); 255.0299(44.6); 227.0349(20.6)
46	13.891	Kaempferol (acetyl)-pentoside	C_22_H_20_O_11_	459.0933	459.0938	1.1	13	459.0970(60.6); 285.0395(10.4); 284.0331(100); 255.0304(36.7); 227.0350(26.1)
49	14.626	Kaempferol *p*-coumaroyl-hexoside (1)	C_30_H_26_O_13_	593.1301	593.1326	4.3	18	593.1359(81.4); 447.0956(6.9); 285.0408(100); 284.0332(52.3); 255.0297(10.0); 227.0346(4.1)
50	14.747	Kaempferol *p*-coumaroyl-hexoside (2)	C_30_H_26_O_13_	593.1301	593.1331	4.3	18	593.1349(100); 447.0958(9.1); 307.0820(6.2); 285.0404(97.7); 284.0313(57.1); 255.0288(9.3)
54	15.612	Kaempferol *p*-coumaroyl Pentoside	C_29_H_24_O_12_	563.1195	563.1219	4.3	18	563.1230(69.8); 285.0406(100); 284.0316(52.3)
58	16.202	Luteolin *p*-coumaroyl-deoxyhexoside	C_30_H_26_O_12_	577.1352	577.1378	4.6	18	577.1384(11.9); 431.1034(1.8); 284.0333(6.1); 285.0409(100); 283.0223(1.8); 257.0469(2.7); 229.0514(2.3)
68	18.075	Kaempferol di-*p*-coumaroyl deoxyhexoside	C_39_H_32_O_14_	723.1719	723.1752	4.5	24	723.1763(2.5); 577.1391(14.5); 559.1232(8.1); 437.1261(48.6); 397.1358(4.8); 285.0404(100); 284.0322(19.1);273.0759(5.7); 187.0395(8.6); 163.0400(19); 145.0295(4.9)
Lignans								
29	5.812	Isolariciresinol hexoside	C_26_H_34_O_11_	521.2028	521.2046	3.4	10	359.1504(2.6); 329.1396(100); 192.0791(3.9); 193.0833(2.8); 175.0760(5.8); 160.0519(3.0)
32	6.722	Neolignan-9′-*O*- rhamnoside isomer 1	C_25_H_34_O_11_	509.2028	509.2053	4.1	9	509.2044(16.9); 491.1926(13.3); 473.1824(27.9); 461.1813(25.9); 367.1395(53.6); 339.1450(6.2); 313.1290(98.3); 179.0712(100); 167.0711(7.2); 161.0611(12.4); 149.0608(27.6); 147.0445(13.3); 134.0373(8.9); 103.0405(8.0)
33	6.722	Cinchonain-I isomer 1	C_42_H_20_O_9_	451.1035	451.1046	2.5	15	451.1083(9.6); 341.0669(100); 299.0551(27.9); 297.0762 (11.7); 281.0460(11.7);217.0131(7.5); 189.0186(15.0); 177.0185(18.2); 161.0246(6.4)
35	6.848	Cinchonain-I isomer 2	C_42_H_20_O_9_	451.1035	451.1047	2.5	15	451.1056(6.3); 341.0674(100); 299.0593(4.7); 297.0762(6.4); 281.0451(12.5); 279.0652(4.7); 231.0288(6.3); 217.0136(9.3); 189.0178(12.5); 177.0193(12.5); 161.0246 (4.7)
36	6.888	Neolignan-9′-*O*- rhamnoside isomer 2	C_25_H_34_O_11_	509.2028	509.2051	4.4	9	509.2071(4.1); 491.1961(18.1); 473.1830(29.1); 461.1830(25.6); 458.1611(6.6); 367.1398(25.2); 313.1299(66.2); 179.0713(100); 167.0704(6.6); 163.0607(14.0); 147.0443(3.4); 149.0609(26.8); 146.0374(18.9); 103.0374(6.6)
47	13.891	4,9,9′-Trihydroxy-3,3′,5′ -trimethoxy-8-*O*-4′ -neolignan-7-*O*-deoxyhexoside	C_27_H_38_O_12_	553.2304	553.2321	5.5	9	553.2291(6.1); 343.1382(100); 328.1140(19.2); 211.0595(2.1); 183.0647(1.5)
48	14.017	9′-Hydroxy-7′-propen-3′,5′-dimethoxyphenyl-3-methoxyphenyl-7,9-propanediol-4-*O*-hexoside	C_27_H_38_O_12_	551.2134	551.2167	6.0	10	551.2184(4.7); 533.2083(4.7); 343.1394(14.1); 328.1161(4.7); 209.0915(100); 194.0579(31.1); 176.0481(8.1)
Fatty acids								
52	15.278	Trihydroxy-octadecadienoic acid	C_18_H_32_O_5_	327.2177	327.2185	2.5	3	327.2187(88.3); 309.2087(3.0); 291.1962(16.4); 229.1448(51.1); 221.1183(15.4); 211.1341(100); 209.1186(7.9); 183.1385(27.6); 171.1026(33.2)
56	16.067	Hydroxyhexadecanoic acid	C_16_H_32_O_4_	287.2228	287.2233	1.8	1	287.2236(100); 285.2077(7.2); 269.2114(8.8)
61	16.512	Dihydroxyoctadecedienoic quinic acid	C_25_H_42_O_9_	485.2756	485.2780	4.9	5	485.2746(4.3); 311.2230(100); 293.2117(5.6); 275.2012(3.7); 223.1703(16.6); 191.0559 (87.9)
62	16.708	Linolenic acid derivative	C_31_H_38_O_6_	505.2596	505.2589	−1.3	13	505.2605(37.2); 277.2176(100); 227.0323(5.2); 152.9955(9.2)
63	16.864	Hydroxyoctadecatrienoic acid	C_18_H_30_O_3_	293.2122	293.2129	2.3	4	293.2111(65.6); 275.2014(82.9); 221.1539(59.0); 211.1338(25.2); 183.1385(100); 171.1016(50.3)
64	16.883	Linoleic acid derivative	C_34_H_44_O_9_	595.2913	595.2915	0.4	13	595.2940(100); 415.2266(6.3); 315.0492(9.5); 279.2332(43.1); 241.0109(17.2); 152.9952(11.5)
65	17.484	15,16-Dihydroxy-9,12-octadienoic acid	C_18_H_32_O_4_	311.2228	311.2232	1.3	3	311.2238(39.4); 293.2125(12.6); 275.2017(9.9); 235.1710(11.1); 253.1804 (3.4); 223.1707(100); 183.0116(2.8)
66	17.738	Linolenic acid glyceryl-tetrahexoside	C_45_H_76_O_24_	999.4654	999.4685	3.1	8	999.4751(100); 837.4302(2.0); 739.2582(12.1); 721.2452(29.1); 559.1919(3.4); 397.1370(4.0); 221.0682(1.3); 119.0353(1.3)
67	17.885	Linolenic acid derivative	C_32_H_41_NO_4_	502.2963	502.2963	0.0	13	502.2958(17.3); 456.1546(6.3); 277.2175(100); 224.0689(6.3)
69	18.826	Linolenic acid glyceryl-dihexoside	C_33_H_56_O_4_	675.3597	675.3629	4.7	6	675.3622(13.0); 415.1454(43.5); 397.1344(100); 379.1210(2.7); 305.0848(7.8); 277.2158(68.9); 253.0904(5.2); 235.0804(18.3), 179.0557(5.2); 161.0451(3.5); 119.0342(7.8); 89.0247(7.8)
Other compounds								
1	0.334	Quinic acid	C_7_H_12_O_6_	191.0561	191.0561	−0.1	2	191.0565(6.7); 173.0455(5.3); 127.0404(15.0); 111.0459(7.9); 93.0356(60.7); 85.0305(100)
2	0.468	Tyrosine hexoside	C_15_H_21_NO_8_	342.1194	342.1183	−3.3	6	252.0855(2.5); 222.0758(2.5); 191.0567(5.0); 180.0664(100); 119.0507(10.25)
10	1.670	Unknown	C_28_H_26_O_16_	617.1148	617.1128	−3.3	16	617.1133(23.8); 455.0806(11.5); 319.0426(100); 297.0605(12.9); 259.0210(4.4); 215.0321(10.2); 201.0160(27.1); 179.0345(10.2); 135.0295(17.7)
19	3.204	Tuberonic acid	C_18_H_28_O_9_	387.1661	387.1666	1.4	5	387.1649(100); 207.1027(32.7);163.1125(8.6); 119.0347(9.7); 101.0252(8.4); 89.0251(14.1)
40	7.742	Hexenyl-3-hydroxy-3-methyl-glutaryl hexoside	C_18_H_30_O_10_	405.1766	405.1775	2.2	4	405.1775(11.4); 343.1799(5.6); 303.1548(5.6); 261.1311(8.6); 179.0545(17.0); 161.0461(14.2); 125.0237(45.7); 101.0240(65.5); 99.0451(100); 89.0246 (34.3)
51	15.240	Unknown	C_20_H_34_O_9_	417.2130	417.2135	1.2	4	417.2147(51.9); 261.1345(44.5); 247.1190(36.1); 243.1268(3.6); 187.0977(100); 173.0821(94.2); 169.0865(11.1); 125.0973(46.1); 111.0818(46.1)
53	15.482	Unknown	C_38_H_52_O_16_	763.3183	763.3207	3.2	13	763.3236(16.7); 343.1403(100); 328.1156(5.8)
55	15.912	Unknown	C_21_H_36_O_9_	431.2287	431.2298	2.7	4	431.2307(26.5); 261.1346(36.6); 187.0976(100); 169.0868(10.3); 125.0972(40.8)
57	16.086	Unknown	C_21_H_36_O_9_	431.2287	431.2299	2.9	4	431.2309(30.4); 261.1338(31.9); 187.0975(100); 169.0865(8.5); 125.0969(41.5)
59	16.261	Saponin isomer 1	C_48_H_76_O_19_	955.4908	955.4948	4.2	11	955.4990(100); 793.4449 (6.8)
60	16.397	Saponin isomer 2	C_48_H_76_O_19_	955.4908	955.4941	4.1	11	955.4990(100); 793.4449 (6.2)

## Data Availability

Data is contained within the article and supplementary material.

## Supplementary Materials

The following are available online at https://www.mdpi.com/article/10.3390/antiox10071140/s1, Figure S1: Proposed fragmentation pathway for compound **12**, Figure S2: TOF-MS/MS spectra of the [M−H]^−^ ions of compounds **32** (A) and **36** (B), tentatively identified as neolignane-9′-*O*-deoxyhexoside are reported with the structures of different fragment ions tentatively characterized. The proposed fragment pathway of much more abundant ions is also reported (C), Figure S3: MS/MS spectral differences of the [M−H]^−^ ions of compound **47** recorded at different Q-TOF parameters: CE −75 V, CES 25 V and DP −90 V (A) or CE −35 V, CES 10 V and DP −70 V (B). The proposed structures of more abundant ions are also reported, Figure S4: TOF-MS/MS spectrum of compound 48, tentatively identified as 9′-hydroxy-7′-propen-3′,5′-dimethoxyphenyl-3-methoxyphenyl-7,9-propanediol-4-O-hexoside and structures of different tentatively characterized fragment ions, Figure S5: TOF-MS/MS spectrum of metabolite **52**, tentatively identified as trihydroxy octadecadienoic acid, whose hypothetical structure and fragmentation pathway was reported in the grey box (the theoretical *m*/*z* value is reported below each structure), Figure S6: TOF-MS/MS spectrum of metabolite **61**, tentatively identified as dihydroxyoctadecedienoic quinic acid, Figure S7: TOF-MS/MS spectrum of compound **69**. The proposed fragmentation pathway of its [M−H]^−^ ion was reported in the grey box (the theoretical *m*/*z* value is reported below each structure), Figure S8: TOF MS/MS spectra of the [M−H]^−^ ions of metabolites **59** (A) and **60** (B); the proposed chemical structures of fragment ions much more abundant are reported with different neutral losses. The fragment ions detected only in one of two spectra are highlighted by red symbols.
