# Supplementary material for: UHPLC-HRMS Analysis of Fagus sylvatica (Fagaceae) Leaves: A Renewable Source of Antioxidant Polyphenols
Source: Antioxidants (Basel). 2021 Jul 19;10(7):1140. doi: 10.3390/antiox10071140 (PMC8301150; doi:10.3390/antiox10071140)

UHPLC-HRMS analysis of *Fagus sylvatica* (Fagaceae) leaf: a renewable source of antioxidant polyphenols

Marialuisa Formato [1], Simona Piccolella[1], Christian Zidorn [2] and Severina Pacifico[2,*]

Supplementary Material

Figure S1 Proposed fragmentation pathway for compound **12**.

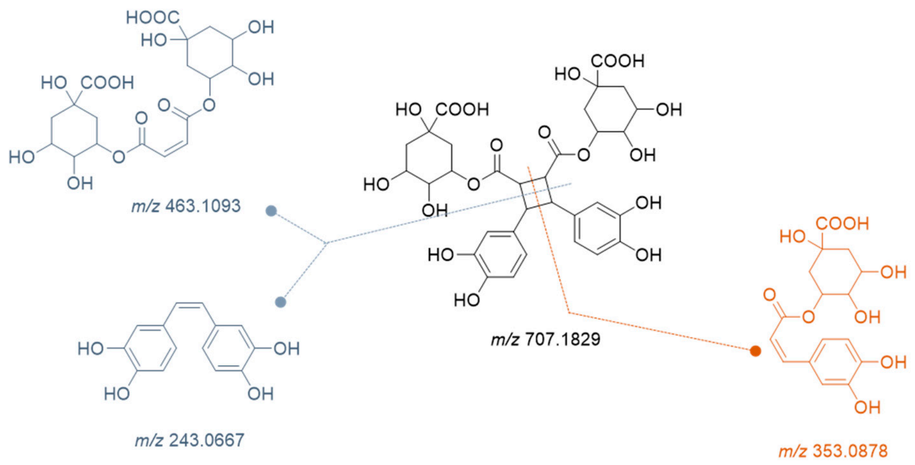

Figure S2 TOF-MS/MS spectra of the [M-H]$^-$ ions of compounds **32** (**A**) and **36** (**B**), tentatively identified as neolignane-9′-*O*-deoxyhexoside are reported with the structures of different fragment ions tentatively characterized. The proposed fragment pathway of much more abundant ions is also reported (**C**).

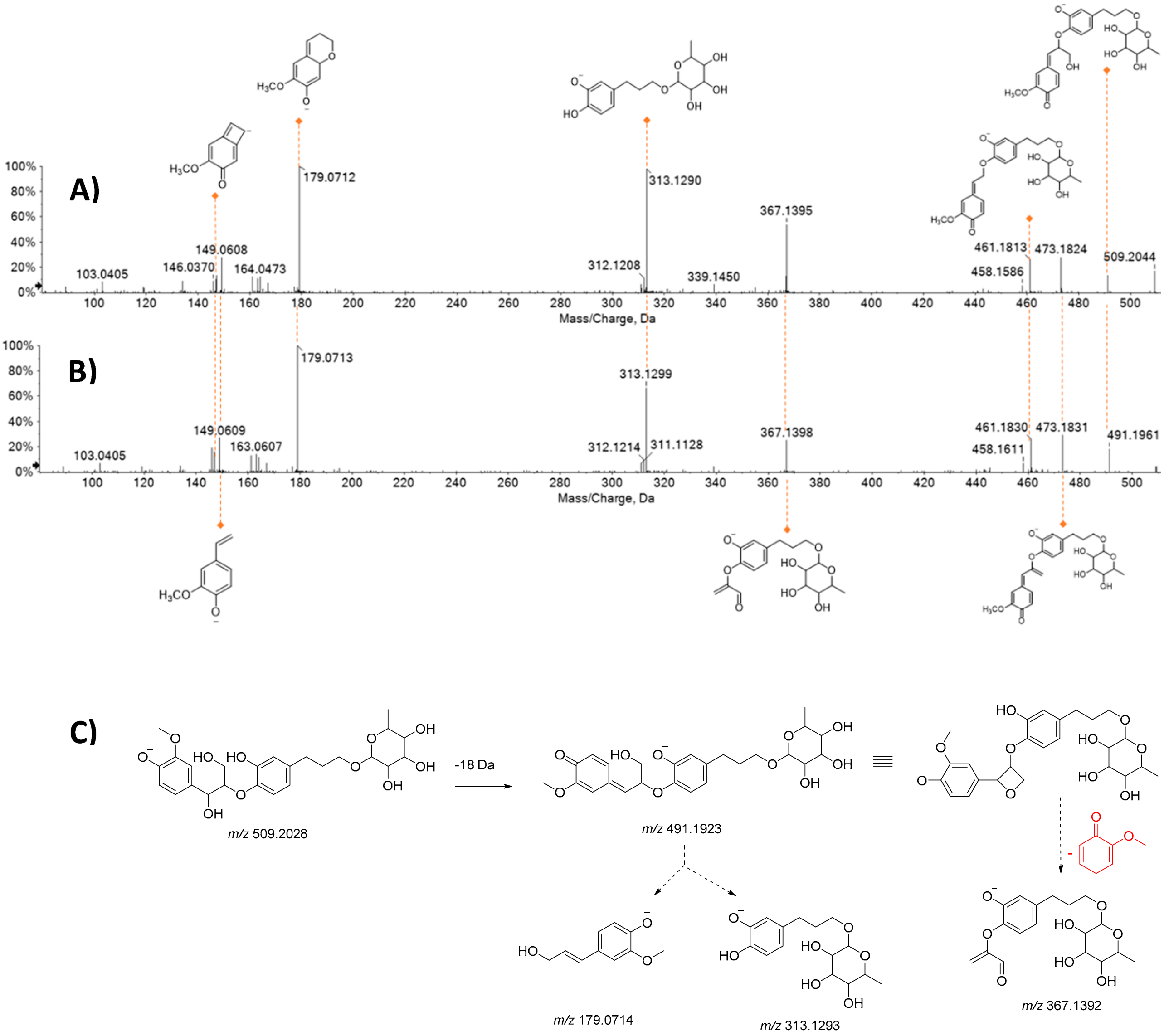

Figure S3 MS/MS spectral differences of the $[M\text{-}H]^-$ ions of compound **47** recorded at different Q-TOF parameters: CE -75 V, CES 25 V and DP -90 V (**A**) or CE -35 V, CES 10 V and DP -70 V (**B**). The proposed structures of more abundant ions are also reported.

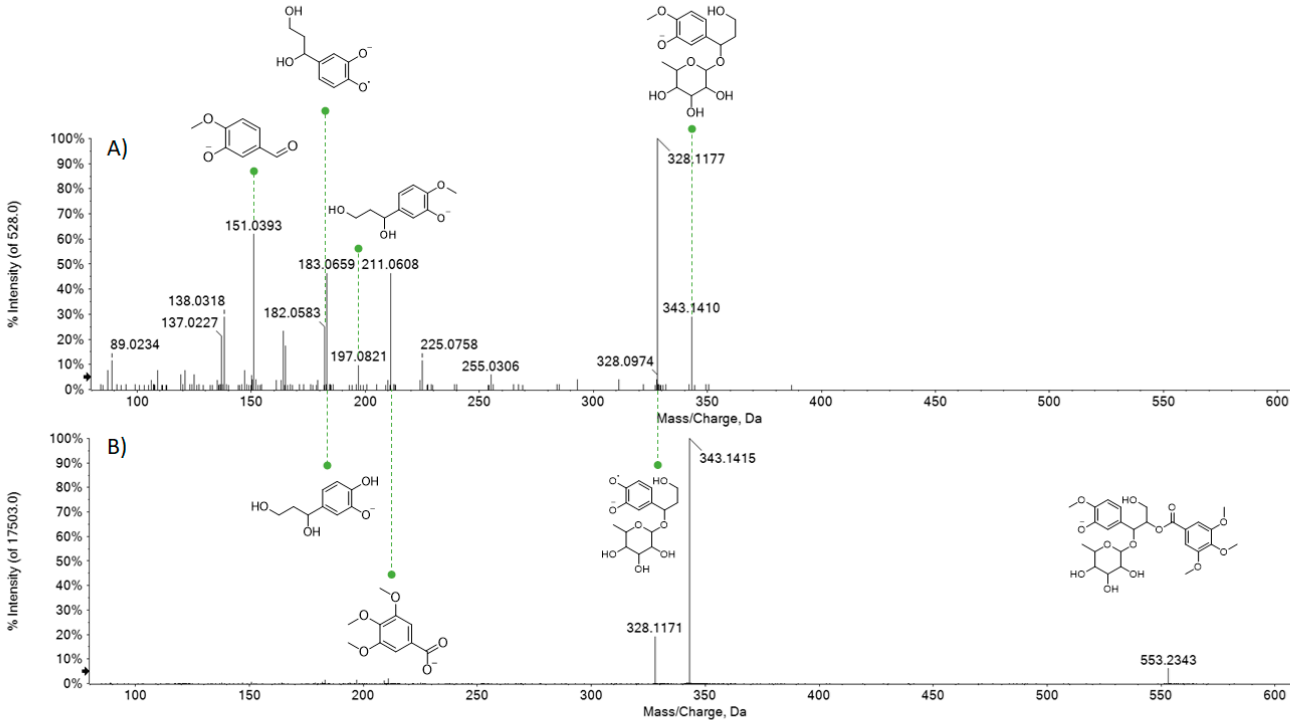

Figure S4 TOF-MS/MS spectrum of compound **48**, tentatively identified as 9′-hydroxy-7′-propen-3′,5′-dimethoxyphenyl-3-methoxyphenyl-7,9-propanediol-4-*O*-hexoside and structures of different tentatively characterized fragment ions.

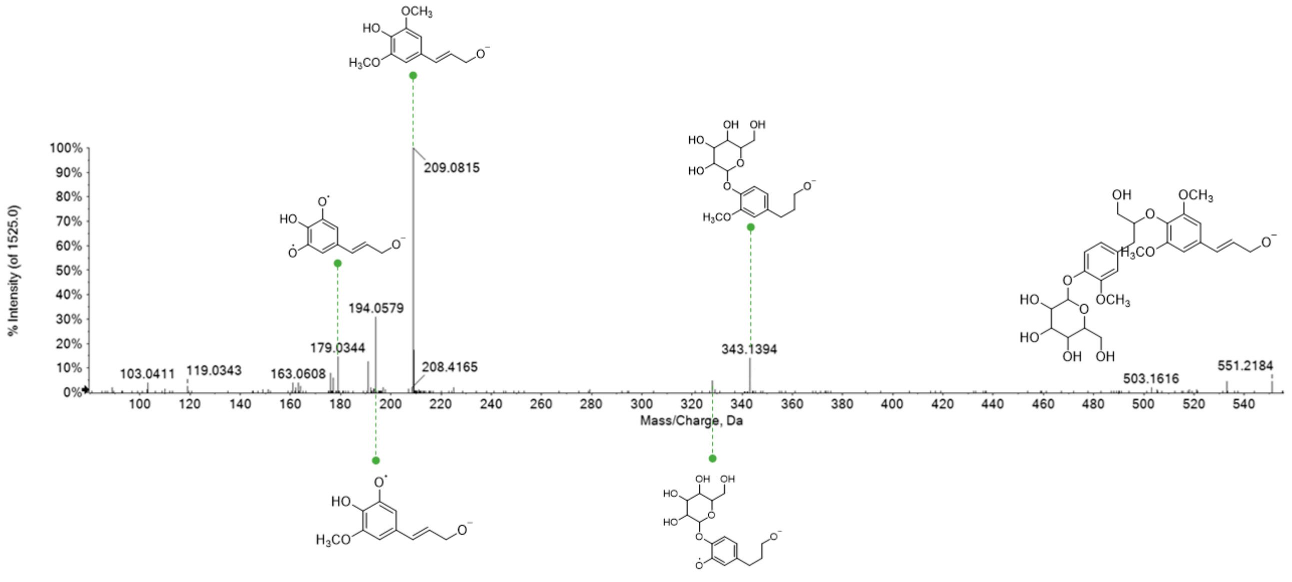

Figure S5 TOF-MS/MS spectrum of metabolite **52**, tentatively identified as trihydroxy octadecadienoic acid, whose hypothetical structure and fragmentation pathway was reported in the grey box (the theoretical *m/z* value is reported below each structure).

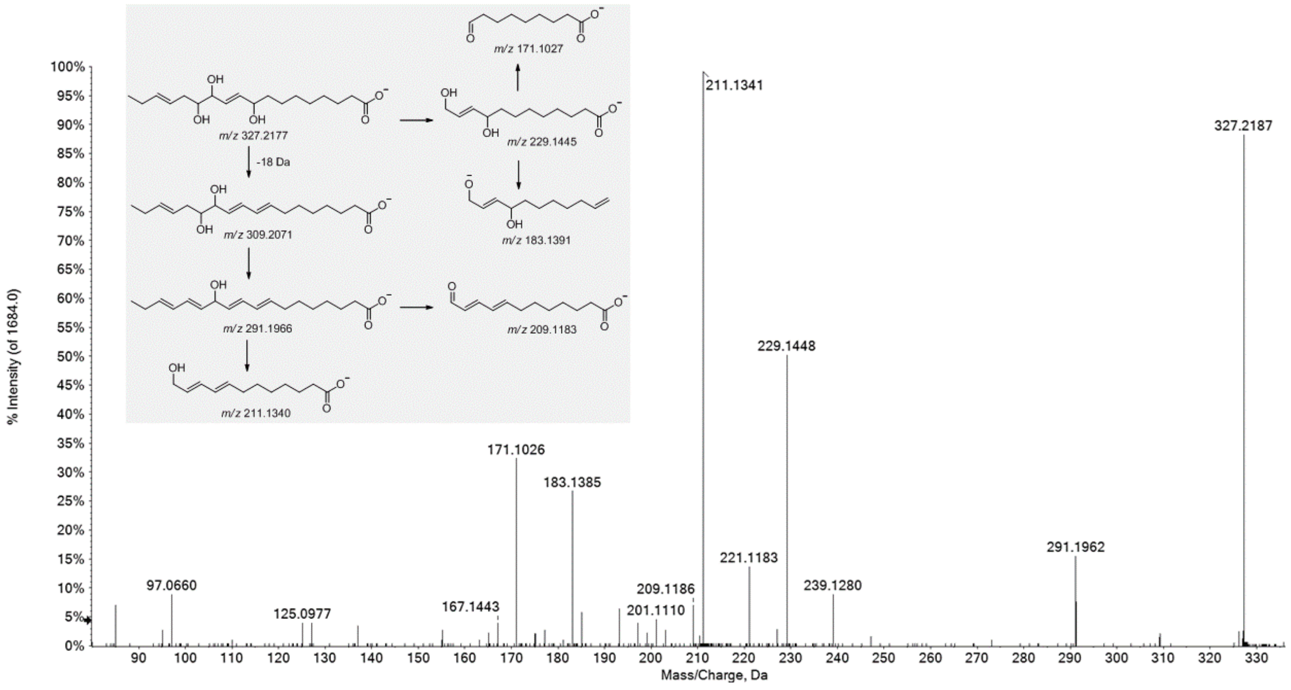

Figure S6 TOF-MS/MS spectrum of metabolite **61**, tentatively identified as dihydroxyoctadecedienoic quinic acid.

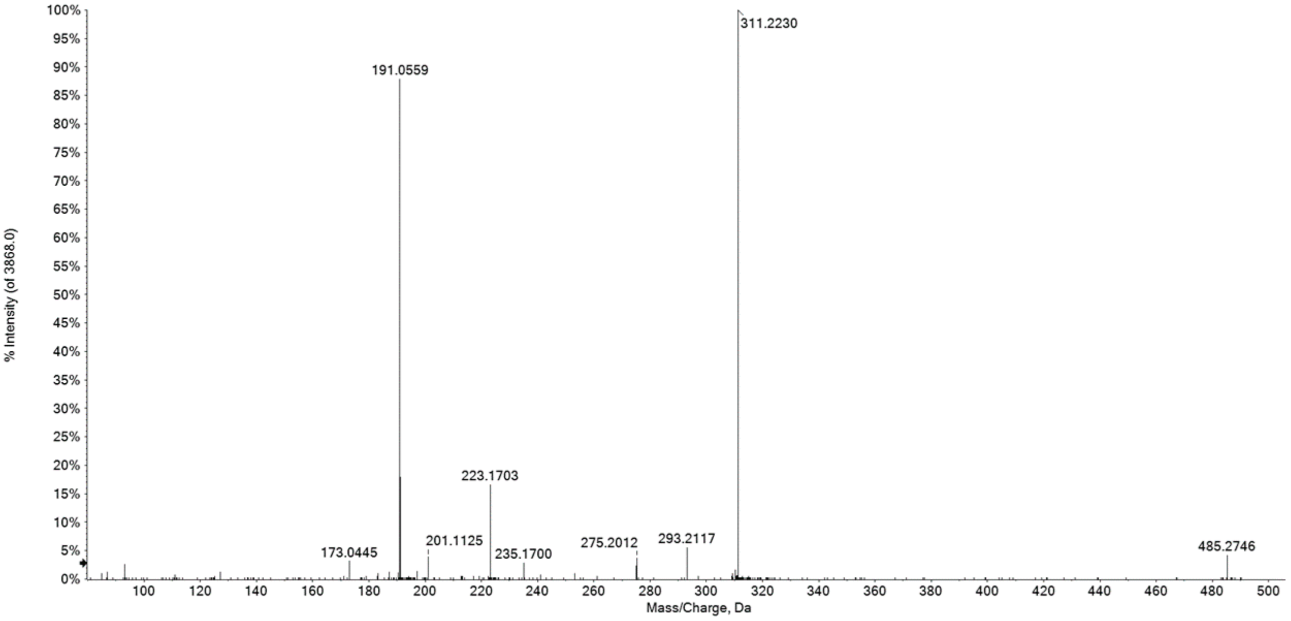

Figure S7 TOF-MS/MS spectrum of compound **69**. The proposed fragmentation pathway of its [M-H]⁻ ion was reported in the grey box (the theoretical *m/z* value is reported below each structure).

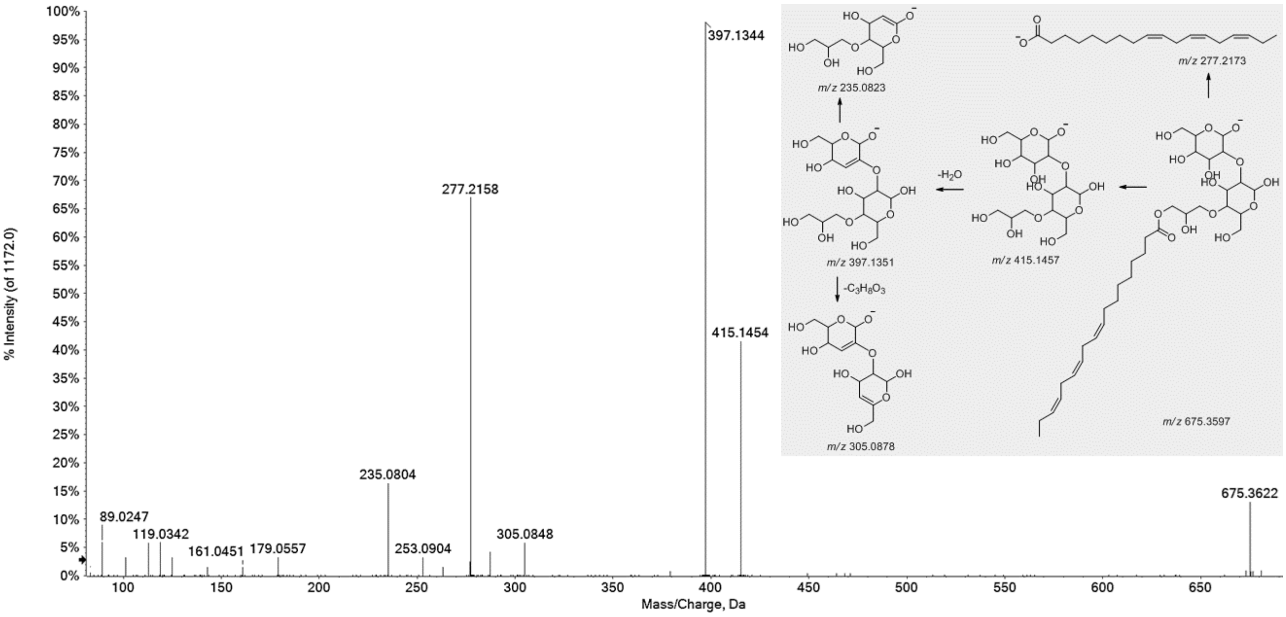

Figure S8 TOF MS/MS spectra of the [M-H]⁻ ions of metabolites **59** (**A**) and **60** (**B**); the proposed chemical structures of fragment ions much more abundant are reported with different neutral losses. The fragment ions detected only in one of two spectra are highlighted by red symbols.

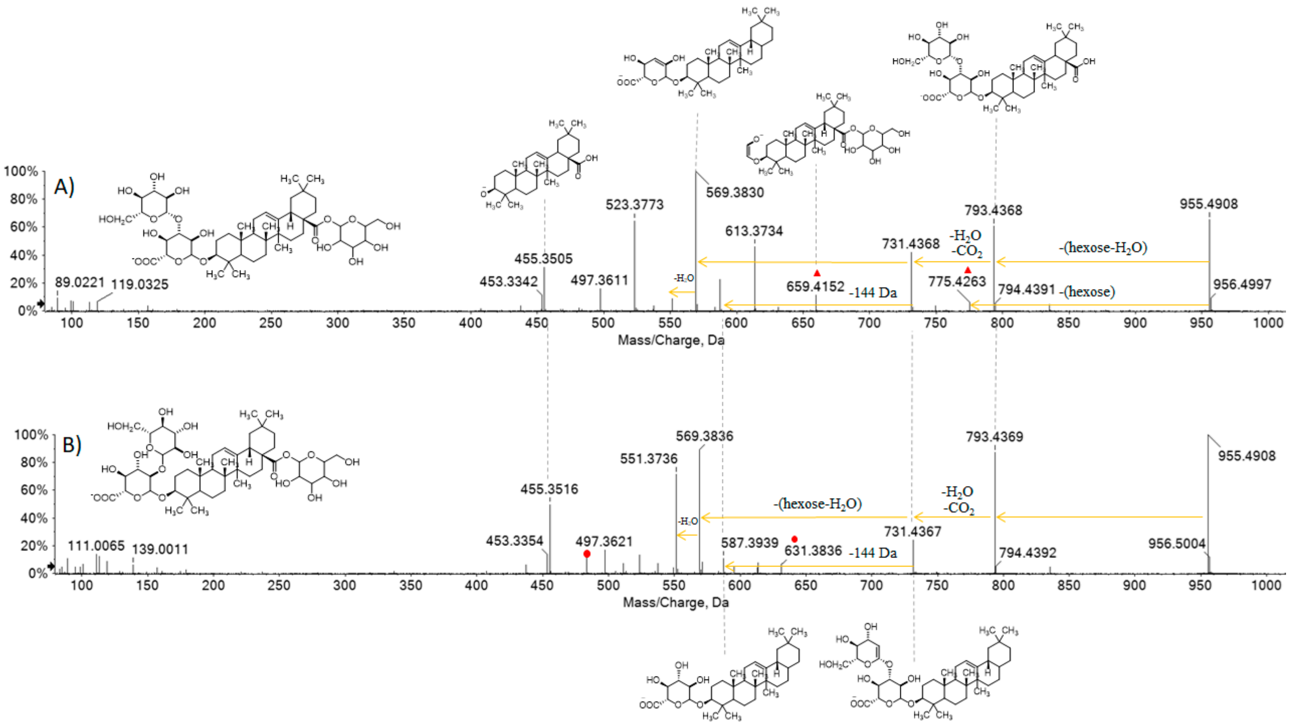

Supplement: Supplementary file 1 [file antioxidants-10-01140-s001.zip › antioxidants-1290887-supplementary.pdf]
